# A Case of Fatal Asthma: Rapid Transition to Cardiac Arrest and Rapid Recovery of Respiratory Status

**DOI:** 10.7759/cureus.11283

**Published:** 2020-11-01

**Authors:** Kasumi Satoh, Manabu Okuyama, Yasuhito Irie, Toshiharu Kitamura, Hajime Nakae

**Affiliations:** 1 Department of Emergency and Critical Care Medicine, Akita University Graduate School of Medicine, Akita, JPN

**Keywords:** out-of-hospital cardiac arrest, post-cardiac arrest syndrome, emergency medicine, asthma, critical care, intensive care units

## Abstract

The global mortality of bronchial asthma continues to decrease, with a reported 57% decline in age-standardized mortality rates between 1993 and 2006.Asthma may rarely be encountered as a cause of cardiac arrest on arrival in the emergency department these days, especially in high-income countries. Rapid worsening of symptoms and rapid improvement in respiratory status after initiation of treatment have been noted as a hallmark of cardiac arrest due to asthma. A 62-year-old male was admitted to our emergency department after dyspnea and dry coughing attack lasting approximately 15 minutes and resulted in cardiopulmonary arrest. His arterial blood gas analysis showed mixed acidosis with pH 7.00, partial pressure of oxygen (PaO_2_) 184, partial pressure of carbon dioxide (PaCO_2_) 90 mmHg, HCO_3_^-^ 22.2, lactate 104 mg/dL. He returned to spontaneous circulation after about 30 minutes of cardiopulmonary arrest. The very slight wheeze on expiration was heard in the left lung and his chest x-ray showed increasing permeability of the lung, which suggested air trapping. Based on his history of asthma, the background of medication discontinuation, and physical findings, the diagnosis of cardiac arrest due to an asthma attack was made. Two hours after admission, PaCO_2_ normalized and his respiratory condition stabilized rapidly. However, epileptic seizures due to hypoxic encephalopathy were prolonged. Although he was then managed in the intensive care unit, he was diagnosed with irreversible brain damage due to hypoxic encephalopathy and shifted to palliative care. Asthmatic cardiac arrest is now rare, but still important. And it may be difficult to identify asthma as a cause of cardiac arrest if the respiratory status improves rapidly. Therefore, keeping in mind the presence of asthma cases of rapid deterioration to cardiopulmonary arrest and case of rapid treatment response may lead to a correct diagnosis.

## Introduction

Asthma is a common non-infectious disease of the lungs. Asthma deaths worldwide are declining due to improvements in therapeutics and are now rare, especially in high-income countries [1]. It is assumed that asthma may rarely be encountered as a cause of cardiac arrest on arrival in the emergency department these days. Rapid worsening of symptoms and rapid improvement in respiratory status after initiation of treatment have been noted as a hallmark of cardiac arrest due to asthma [2]. However, this is only based on a very small observational study. We describe a case of cardiac arrest due to asthma, which is rarely seen nowadays. We also detail the rapidly progressive nature of the attacks and the rapid response to treatment.

## Case presentation

A 62-year-old male was admitted to our emergency department (ED) after dyspnea and dry coughing attack lasting approximately 15 minutes, followed by pallor, loss of consciousness, and collapse. He was in cardiopulmonary arrest with pulseless electrical activity at the time of paramedic contact. He had been under treatment for bronchial asthma and had been prescribed fluticasone and salmeterol oral inhalation, however stopped treatment a month before due to financial problems. He had occasional nocturnal asthma attacks, however, they were self-limited, hence he was tolerated without a hospital consultation. Meanwhile, he smoked 10 cigarettes per day.

**Figure 1:**
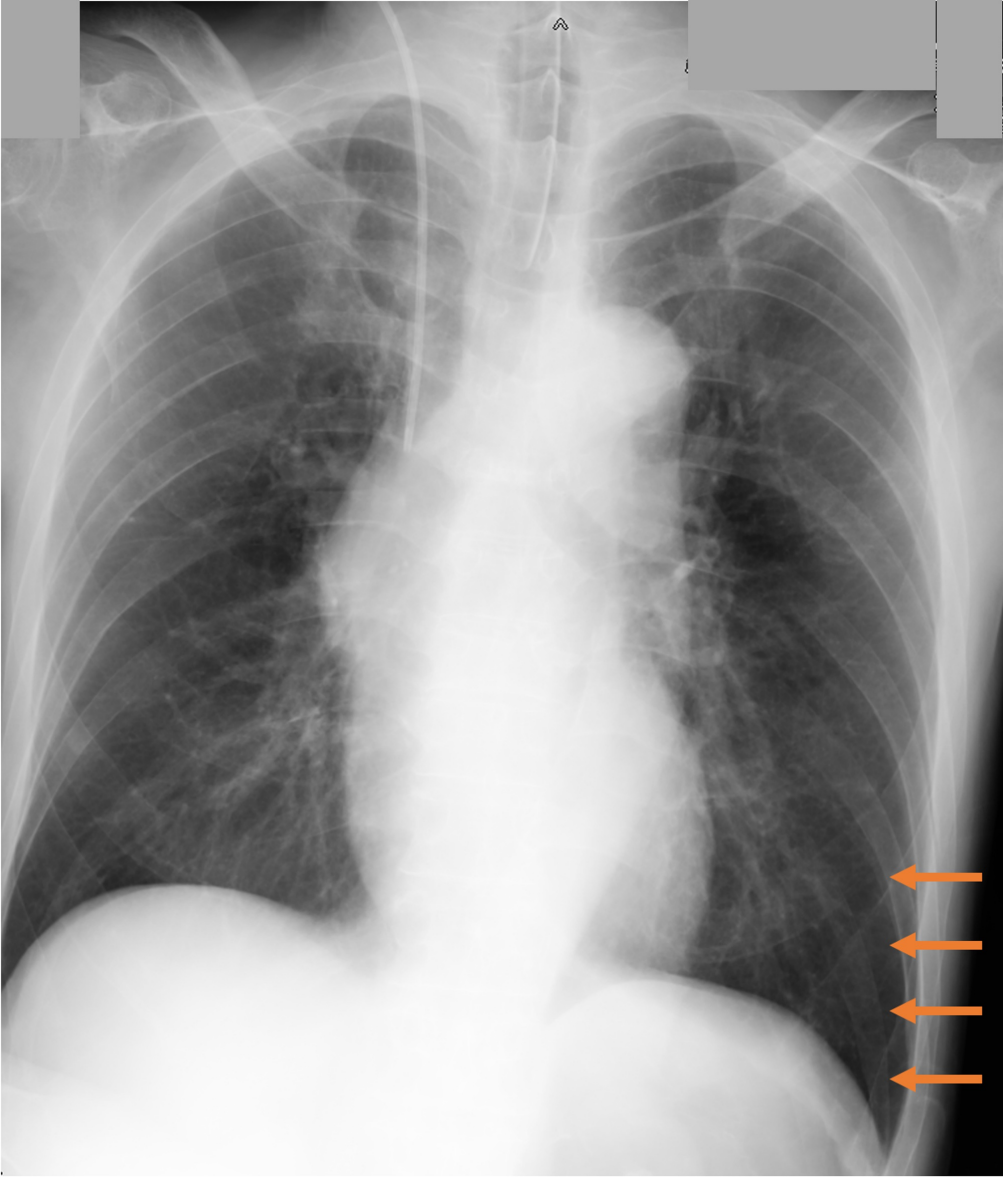
A supine chest x-ray in the emergency department His supine chest x-ray showed slightly increased permeability of the lung (orange arrow), which suggested air trapping.

On our examination in the ED, he remained in cardiopulmonary arrest with pulseless electrical activity. Arterial blood gas analysis showed mixed acidosis with pH 7.00, partial pressure of oxygen (PaO_2_) 184 mmHg, partial pressure of carbon dioxide (PaCO_2_) 90 mmHg, lactate 104 mg/dL, HCO3^-^ 22.2, base excess -10.9 mmol/L. We performed advanced cardiovascular life support, i.e. chest compressions, tracheal intubation, ventilator induction, and 1 mg of adrenaline, then he returned of spontaneous circulation (ROSC) after about 30 minutes of cardiopulmonary arrest. Immediately after ROSC, his Glasgow Coma Scale (GCS) was 5 points (E1V1M3), his bilateral pupil diameter was 3 mm and his direct contralateral light reflex was slow. His spontaneous breathing was weak and a very slight wheeze on expiration was heard in the left lung. His supine chest x-ray (Figure 1) did not show any abnormal shading in the lung fields, meanwhile there was increased permeability of the lung, which suggested air trapping. His laboratory blood tests, plain head computed tomography (CT), trunk contrast CT, and electrocardiography performed in the ED did not show any findings indicating the cause of cardiac arrest. Based on his history of asthma, the background of medication discontinuation, and physical findings, the diagnosis of cardiac arrest due to an asthma attack was made. He was given intravenous diazepam and continuous intravenous midazolam in the ED for a variety of involuntary movements, including brief clonic seizures and jerking of the extremities.

**Figure 2:**
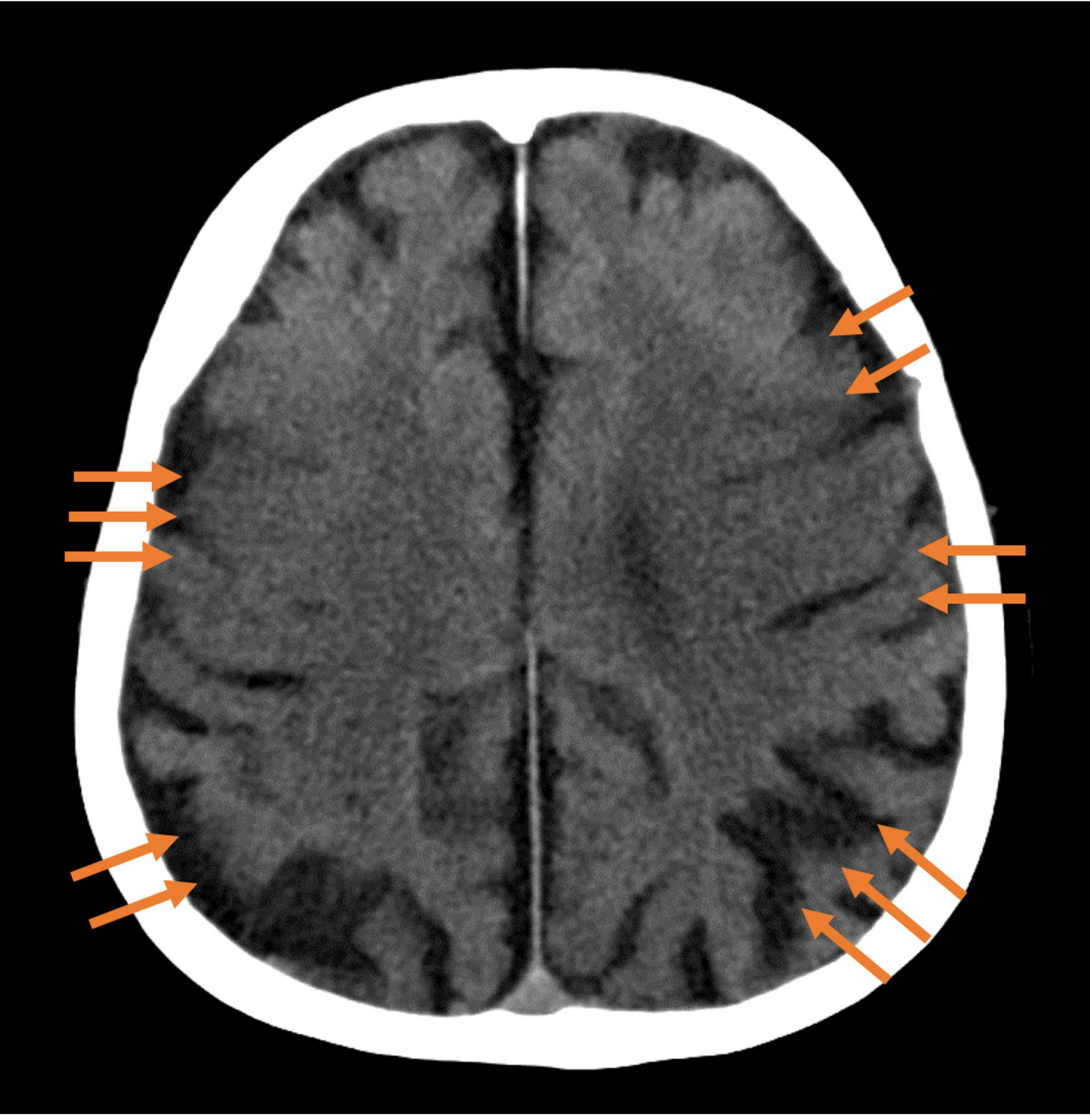
A plain computed tomography scan of the head taken on the fourth day of hospitalization A plain computed tomography scan  showed the disappearance of grey-white differentiation, suggesting hypoxic encephalopathy

He was admitted to the intensive care unit where he was treated with temperature control therapy (24 hours at 35°C) and ventilator management. Arterial blood gas analysis two hours after the patient's arrival showed a pH of 7.37, PaO_2_ 42 mmHg, HCO3^-^ 20.2, and lactate 79 mg/dL; normalization of carbon dioxide was quickly achieved, with a PaO_2_: fraction of inspired oxygen (FiO_2_) ratio of 223. A slight extension of exhalation time was recognized for several hours. Methylprednisolone 40 mg was administered intravenously as a control for asthma. Although his respiratory status quickly stabilized, his epilepsy was prolonged despite the administration of intravenous fosphenytoin, enteral levetiracetam and clonazepam, and continuous intravenous propofol and midazolam. The antiepileptics were reduced after control of the seizure, however, he never recovered from GCS 3 points (E1V1M1). A plain CT scan of the head taken on the fourth day of hospitalization showed the disappearance of grey-white differentiation (Figure 2). We diagnosed him with irreversible brain damage due to hypoxic encephalopathy and shifted him from intensive care to palliative care. He was transferred to the general ward on day eight and then passed away on day 56.

## Discussion

We identified two important clinical issues: (1) while asthma as a cause of cardiac arrest is becoming rarer, it can be encountered in the emergency room, and (2) some fatal asthma that leads to rapid cardiac arrest may rapidly stabilize the respiratory state, and the patient may have few asthma features immediately after resuscitation.

First, even though cardiac arrest due to asthma is rare, we may face it in the emergency room. The Global Burden of Disease reports that bronchial asthma is responsible for 420,000 deaths per year worldwide, with many avoidable causes (e.g. poor access to medical care) [3]. However, global mortality continues to decline due to improvements in treatment, with a reported 57% decline in age-standardized mortality rates between 1993 and 2006 [4]. Asthma is no longer a common cause of death, especially in higher-income countries. For instance, according to the Centers for Disease Control and Prevention (CDC), the number of deaths from asthma in the United States in 2016 was reported to be very low at 10 per million people [1]. This is a valuable report describing cardiac arrest due to asthma, which continues to decrease. Because of its rarity but severity, it is recommended that asthma be kept in mind as a cause of cardiac arrest.

Second, some fatal asthma that leads to rapid cardiac arrest can rapidly stabilize the respiratory state. We consider that the adrenaline used for resuscitation and the sedative drugs used after resuscitation may affect rapid respiratory stability. Immediately after resuscitation, the respiratory status may recover enough to lack almost all of the features of asthma that asthma can be difficult to recognize as a cause of cardiac arrest in this case. Plaza et al. [5] reported that about 20% of patients with fatal and near-fatal asthma had rapid-onset asthma, and the baseline characteristics of patients with rapid-onset asthma were no different from those without. Rapid-onset asthma was found the trigger in half of the patients, and fume/irritant inhalation and intake of nonsteroidal anti-inflammatory drugs are characterized as triggers [5], but there were no triggers in this case. Kolbe et al. [6] showed that 8% of patients with asthma requiring hospitalization were rapid-onset asthma, and severe cases, including those with cardiac arrest and ventilator induction, were significantly more frequent in the rapid-onset asthma group. It has been noted that patients with rapid onset attacks often recover very quickly [6], and Plaza et al. [5] and Wasserfallen et al. [7] also suggested patients with rapid-onset asthma had a more rapid response to treatment to mechanical ventilation. Kataoka et al. [2] reported that asthma patients who developed cardiac arrest were characterized by a shorter time between symptom onset and transition to ventilator management and a shorter time to normalization of PaCO_2_ compared to near-fatal asthma patients. In this report, the authors assumed that asthma attacks in cases of cardiac arrest cases have a specific pathophysiological feature such as reversible airway constriction rather than airway obstruction due to mucus plugs [2]. In the present case, PaCO_2_ was as high as 90 mmHg at the time of arrival to ED, which was halved and normalized approximately two hours later, consistent with the time course in this study. In this case, we diagnosed asthma as the cause of the cardiac arrest through the background of self-discontinuation of asthma medication, hearing of a very faint wheeze from the left lung after resuscitation and prolonged expiration, and denial of other diseases. The wheeze quickly disappeared, and its extended exhalation also disappeared in a few hours. Without careful observation, the diagnosis of asthma would not have been easy to make. Detailed patient examination and identification of the background that causes severe asthma will help make a diagnosis. And above all, remembering the presence of an asthmatic cardiac arrest, which stabilizes the respiratory state immediately, helps in the correct diagnosis. There does not appear to be any detailed description or large-scale study literature on the trajectory of rapid onset asthma attacks leading to cardiac arrest. Therefore, we regard our present case report as significant.

## Conclusions

In conclusion, asthmatic cardiac arrest is rare today, however, it's still important as clinicians can face it. Fatal asthma is associated with a relatively high rate of rapid deterioration. And after resuscitation of cardiac arrest, relatively rapid response to treatment has been suggested. In some instances, as in the current case, there may be only a few signs of asthma remain after resuscitation. Being aware of the trajectory that asthmatic cardiac arrest can be rapidly recovered will bring about the correct diagnosis.
